# Ultrasound‐derived velocity variations predict fluid responsiveness in critically ill pediatric oncology patients

**DOI:** 10.14814/phy2.70756

**Published:** 2026-02-03

**Authors:** Bruno Sanchez Camargo, Orlei Ribeiro de Araujo, Dafne Cardoso Bourguignon da Silva

**Affiliations:** ^1^ Pediatric Intensive Care ‐ Support Group for Adolescents and Children with Cancer (GRAACC)/Institute of Pediatric Oncology/Federal University of São Paulo (UNIFESP) São Paulo Brazil

**Keywords:** blood volumes, cancer care unit, child, criticall illness, diagnostic ultrasound, echocardiography

## Abstract

Dynamic ultrasound indices assess preload‐dependent changes in stroke volume via the Frank–Starling mechanism and guide fluid therapy. This study aimed to determine optimal cutoff values for ultrasound‐derived peak aortic (ΔVAo) and carotid (ΔVCa) velocity variations to predict fluid responsiveness in critically ill pediatric oncology patients. In this prospective cohort, 83 children underwent 88 fluid challenges with 10 mL/kg saline. Fluid responsiveness was defined as a >15% increase in cardiac index, measured by left ventricular outflow tract Doppler after volume expansion. Fluid responsiveness occurred in 54.5% of assessments. ΔVAo demonstrated the highest predictive accuracy, with a 16.3% cutoff (sensitivity 91.6%, specificity 80%, AUC 0.89). ΔVCa showed moderate performance (cutoff 14.2%; sensitivity 79.1%, specificity 65%, AUC 0.75), while ΔIVC was not predictive (AUC 0.56). In mechanically ventilated patients (*n* = 60), ΔVAo remained accurate (cutoff 16.3%; AUC 0.90), whereas ΔVCa was modest (cutoff 16.5%; AUC 0.74). In spontaneously breathing patients (*n* = 28), ΔVAo cutoff was 15.5% (sensitivity 95%, specificity 87.5%, AUC 0.89), and ΔVCa was 13.2% (sensitivity 100%, specificity 50%, AUC 0.69). ΔVAo is a reliable predictor of fluid responsiveness in critically ill pediatric oncology patients. ΔVCa may serve as an alternative, though with lower accuracy.

## 1 INTRODUCTION

Fluid management in critically ill children presents a significant clinical challenge. Both hypovolemia and fluid overload are associated with serious complications, including organ dysfunction, increased morbidity, and mortality. Therefore, accurately identifying fluid responsiveness—determining which patients will benefit from additional fluid administration—is essential for guiding effective resuscitation strategies in pediatric intensive care units (Cecconi et al., 2014; Malbrain et al., 2018).

Fluid responsiveness reflects a physiological state in which the heart operates on the ascending limb of the Frank‐Starling curve, meaning that an increase in preload leads to a meaningful rise in stroke volume and cardiac output (Malbrain et al., 2018). Traditional static parameters such as central venous pressure, blood pressure, and heart rate have shown limited reliability in predicting this response, as they fail to capture dynamic changes in cardiac preload (Gan et al., 2013). In contrast, dynamic indices that assess the heart's response to transient preload variations offer superior predictive accuracy. A patient can be considered as fluid responsive if cardiac output increases by at least 15% following a fluid challenge (Boyd et al., 2016).

Point‐of‐care ultrasound (POCUS) has emerged as a valuable, noninvasive modality for bedside hemodynamic assessment in critically ill pediatric populations (Carioca et al., 2023; Levitov & Marik, 2012). Dynamic ultrasound variables, particularly those measuring changes in peak aortic (ΔVAo) and carotid (ΔVCa) velocities, provide a practical approach to evaluating fluid responsiveness. However, specific cutoff values for these parameters remain poorly defined in certain subgroups—especially in critically ill children with cancer.

This study aims to establish optimal ultrasound‐derived cutoff values for ΔVAo, ΔVCa, and respiratory variation of the inferior vena cava (ΔIVC) to accurately predict fluid responsiveness in this vulnerable patient cohort.

## 2 METHODS

This prospective interventional cohort study was conducted in the oncology intensive care unit (ICU) of a specialized pediatric cancer center in São Paulo, Brazil, between July 2023 and March 2025. Ethical approval was granted by the Research Ethics Committee of the Federal University of São Paulo (Certificate No. 68803323.9.0000.5505, approved July 19, 2023), in accordance with Resolution 196/96 of the National Health Council and the principles of the 1975 Declaration of Helsinki. The research protocol is registered on the Plataforma Brasil (http://plataformabrasil.saude.gov.br). Written informed consent was obtained from the legal guardians of all participants prior to study inclusion.

Eligible patients were children and adolescents under 18 years of age with a cancer diagnosis and critical condition, requiring volume expansion as determined by the attending physician. Inclusion criteria included central venous access for blood collection and signed informed consent. Exclusion criteria were inadequate transthoracic echocardiographic windows, congenital heart disease, left ventricular ejection fraction <50%, severe arrhythmias, or signs of pulmonary congestion on initial extravascular lung water (EVLW) assessment—defined as more than two B‐lines per intercostal space or confluent B‐lines. Lung ultrasound was performed to assess pulmonary fluid tolerance and detect early signs of fluid overload following volume expansion. Given the vulnerability of pediatric oncology patients to capillary leak and pulmonary complications, lung ultrasound allowed us to complement hemodynamic assessment with safety monitoring.

Patients received a 10 mL/kg saline bolus (maximum 500 mL), followed by reassessment (30–60 min). Fluid boluses were administered over approximately 15–20 min, in accordance with local pediatric intensive care practice and patient safety considerations.

Baseline demographic data were collected, and central venous blood gas analysis, echocardiography, carotid Doppler, and lung ultrasound were performed before and after volume administration.

**FIGURE 1:**
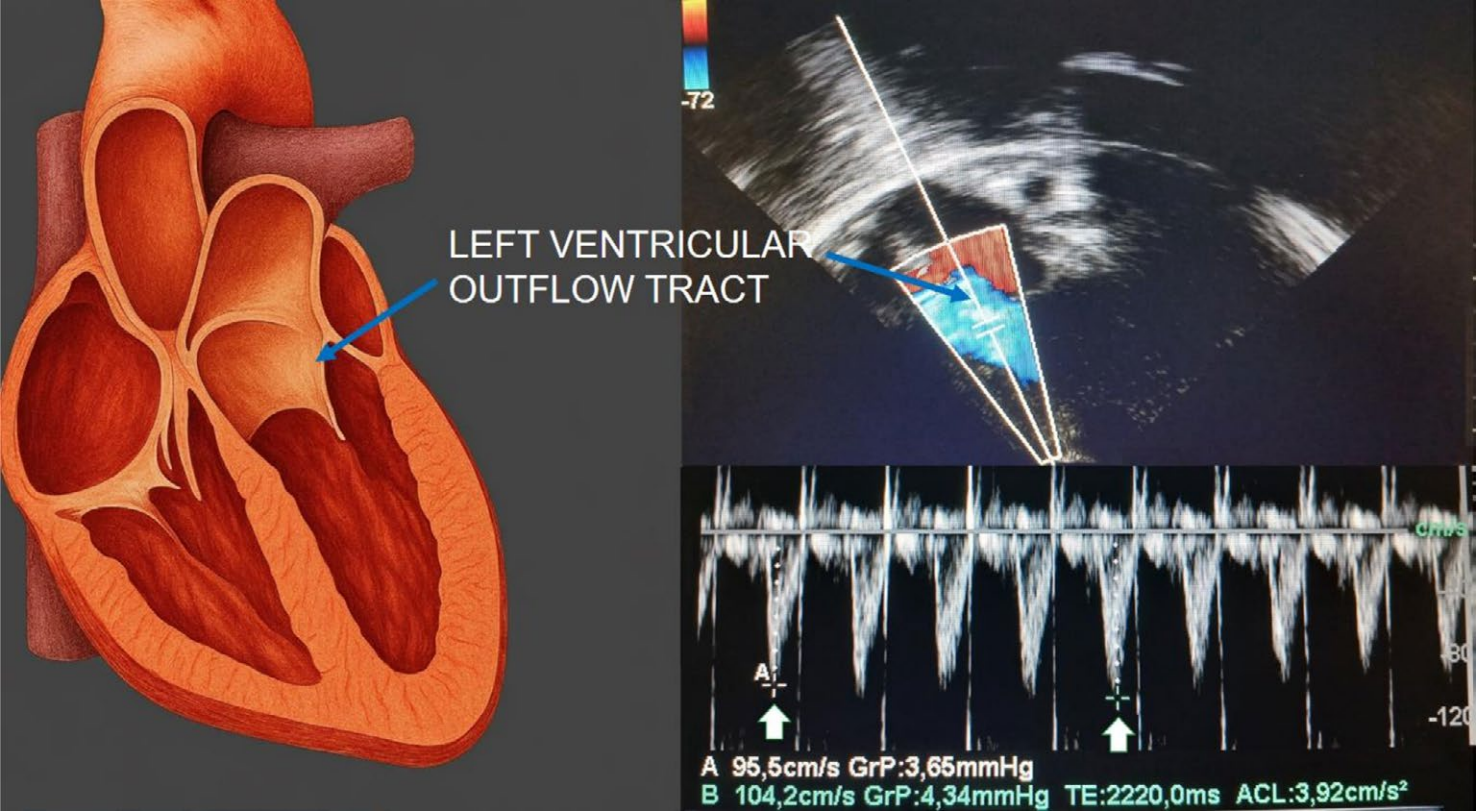
Method of measuring aortic flow velocities, with pulsed Doppler placed in the left ventricular outflow tract.

Ultrasound assessments were performed by a trained investigator using Sonosite Turbo® equipment. Respecting patient comfort and to avoid excessive manipulation, the assessments were not performed in duplicate. Lung ultrasound was performed with a linear transducer (13–6 MHz frequency band), evaluating 12 thoracic areas. Carotid Doppler used a linear probe with a 30°–60° angle and a 0.3–0.5 mm sampling gate (Lee, 2014). Echocardiography utilized a 5–1 MHz cardiac transducer to measure cardiac output via left ventricular outflow tract (LVOT) Doppler, following Mclean et al. methodology (McLean et al., 1997). Transthoracic echocardiography was used as the reference method to define fluid responsiveness. The LVOT diameter was measured between the bases of the aortic valve leaflets during systole, as observed in the long parasternal view. Pulsed Doppler samples were obtained in the center of the LVOT from the apical view, with an angle of the Doppler signal relative to the aortic blood flow <20° (Mercado et al., 2017). Figure 1 illustrates the method for measuring the variation in aortic flow velocities. A positive fluid response was defined as an increase greater than 15% in cardiac index measured by left ventricular outflow tract Doppler following the fluid challenge.

ΔVAo and ΔVCa were calculated as: Δ = (max−min)/((max + min)/2) × 100. The inferior vena cava collapsibility index was calculated as: ΔIVC = (inferior vena cava maximum diameter−minimum diameter/maximum diameter) × 100.

Statistical analysis included descriptive statistics, Wilcoxon tests for paired comparisons, and rank‐biserial correlation to assess effect size (small <0.3, medium 0.3–0.5, and large >0.5) (Kerby, 2014). Receiver Operating Characteristic (ROC) curves were used to determine optimal cutoff values for ΔVAo, ΔVCa, and ΔIVC, with sensitivity, specificity, and accuracy calculated. Analyses were conducted using jamovi (v2.6) and R software.

## 3 RESULTS

During the study period, 786 patients were admitted to the pediatric ICU. A total of 88 fluid responsiveness assessments were conducted in 83 eligible patients, with some undergoing repeated evaluations during separate ICU admissions. Clinical and demographic characteristics are summarized in Table 1.

**TABLE 1:** Baseline characteristics of included participants (*N* = 83).

Data		
Gender, male, (*N*, %)	46	55.4%
Age in months (median, IQR)	69	39–100.5
Cancer diagnoses (*N*, %)		
Acute leukemia (lymphoblastic and myeloid, Burkitt's leukemia)	16	19.2%
Ewing's sarcoma	4	4.8%
Glioma	5	6.0%
Lymphoma (Hodgkin's and Non‐Hodgkin's)	4	4.8%
Medulloblastoma	9	10.8%
Neuroblastoma	7	8.4%
Other central nervous system tumors	15	18.1%
Other neoplasms	16	19.3%
Teratoid rhabdoid tumor	5	6.0%
Wilms tumor	2	2.4%
Causes of admission (*N*, %)		
Acute obstructive abdomen	2	2.4%
Acute respiratory failure	11	13.3%
Decreased level of consciousness	3	3.6%
Hyperleukocytosis	2	2.4%
Intracranial hypertension	2	2.4%
Other diagnoses	10	12.0%
Other types of shock	9	10.8%
Post‐operative of neurosurgery/orthopedics/pediatric surgery	18	21.7%
Seizures	4	4.8%
Sepsis without shock	3	3.6%
Septic shock	19	22.9%
Upper gastrointestinal bleeding	3	3.6%
Hematopoietic Stem Cell transplantation (*N*, %)	19	22.9%
PRISM IV raw score (mean, SD)	11.1	7.7
Vasoactive drugs	24	28.9%
Number of B‐lines per intercostal space, pre‐volume (mean, SD)	1.42	1.13
In‐hospital mortality	23	27.7%
Mechanical ventilation	60	68.1%

Abbreviations: IQR, interquartile range 25–75; SD, standard deviation.

Fluid responsiveness was observed in 48 assessments (54.5%). Among responders, mean CI increased from 3.28 L/min/m^2^ (SD 0.93) to 4.19 L/min/m^2^ (SD 1.16) following volume expansion (*p* < 0.001; effect size = 1). Aortic velocity‐time integral (VTI) increased in 75 assessments (85.2%), from a mean of 15.5 (SD 3.41) to 17.7 (SD 3.93; *p* < 0.001; effect size = 0.8). Additional hemodynamic and laboratory changes are detailed in Table 2.

**TABLE 2:** Observed effects of volume bolus on hemodynamic and laboratory parameters, pre‐ and post‐ volume expansion.

	Responders				Nonresponders			
	Mean	SD	*p* Value (pre‐post)	Effect size	Mean	SD	*p* Value (pre‐post)	Effect size
ΔVAo pre (%)	27.9	13.3	<0.001	−0.8	13.3	7.03	0.13	–
ΔVAo post (%)	17.3	10.7			15.1	7.37		
ΔVCa pre (%)	22.6	10.1	<0.001	−0.54	14.7	8.23	0.22	–
ΔVCa post (%)	17	11.2			16.5	9.38		
ΔVCI pre (%)	35.8	18.5	0.44	–	31.6	17.7	0.04	−0.37
ΔVCI post (%)	33.7	18.6			26	16.2		
LV ejection fraction pre (%)	66.1	8.55	0.03	0.39	67.8	10.2	0.73	–
LV ejection fraction post (%)	67.7	7.79			66.9	10.2		
Heart rate pre (bpm)	117	28.7	0.77	–	116	28.2	0.014	−0.46
Heart rate post (bpm)	118	28.5			113	29		
pH pre	7.33	0.07	0.6		7.34	0.07	0.9	–
pH post	7.33	0.07			7.34	0.07		–
CO_2_ pre (mmHg)	44.9	8.9	0.08	‐	47.9	11.8	0.59	–
CO_2_ post (mmHg)	44	9.4			47.3	11.8		
Bicarbonate pre (mmol/L)	23.2	5.6	0.016	−0.4	24.4	5.17	0.9	–
Bicarbonate post (mmol/L)	22.4	5.1			24.4	4.8		
Lactate pre (mmol/L)	1.7	1.7	0.18	–	1.3	1.09	0.023	−0.44
Lactate post (mmol/L)	1.71	1.8			1.15	1.21		
Hemoglobin pre (g/dL)	9.58	2.48	0.62	–	9.53	1.97	0.22	–
Hemoglobin post (g/dL)	10	2.21		–	9.85	2.35		
Venous oxygen content pre (mL/dL)	9.86	3.7	0.67	–	9.23	3.23	0.53	–
Venous oxygen content post (mL/dL)	10.3	3.1		–	9.84	3.08		–
Sodium pre (mmol/L)	143	7.77	0.23	–	140	6.5	0.29	–
Sodium post (mmol/L)	144	5.98			141	6.7		
Chloride pre (mmol/L)	109	8	0.004	0.56	105	10	0.08	–
Chloride post (mmol/L)	110	8.1			106	9.6		

Abbreviations: LV: left ventricle; SD, standard deviation.

Receiver Operating Characteristic (ROC) analysis for the total sample identified ΔVAo as the strongest predictor of fluid responsiveness, with a cutoff of 16.3% determined by the Youden index, sensitivity of 91.6%, specificity of 80%, AUC of 0.89 (95% CI: 0.83–0.97), and accuracy of 86%. The positive likelihood ratio (LR+) was 4.58, and the negative likelihood ratio (LR‐) was −0.14, with a positive predictive value (PPV) of 0.84 and a negative predictive value (NPV) of 0.89. ΔVCa had a cutoff of 14.2%, sensitivity of 79.1%, specificity of 65%, AUC of 0.75 (95% CI: 0.64–0.86), and accuracy of 72%. The LR+ was 2.26, and the LR− was −0.21. The PPV was 73%, and the NPV was 72%. ΔIVC showed poor predictive value (AUC 0.56; 95% CI: 0.44–0.68), for a cutoff point of 39.6%.

In mechanically ventilated patients (*n* = 60), ΔVAo decreased from 19.5% (SD 12.4) to 15.3% (SD 8.7; *p* = 0.012; effect size = −0.37) following expansion. ΔVCa showed a nonsignificant reduction (17.9%–16%; *p* = 0.16), while ΔIVC decreased from 34.3% to 28.4% (*p* = 0.029; effect size = −0.33). Volume responsiveness was observed in 28 cases (46.6%). ΔVAo retained its cutoff at 16.3%, with sensitivity of 92.8%, specificity of 78.1%, AUC of 0.90 (95% CI: 0.82–0.98), and accuracy of 85%. The LR+ was 4.23, the LR− was 0.18, the PPV was 78%, and the NPV was 92.7%. ΔVCa had a cutoff of 16.5%, sensitivity of 75%, specificity of 78.1%, AUC of 0.74 (95% CI: 0.62–0.88), and accuracy of 76%. The LR+ was 3.4, the LR− was 0.038, the PPV was 74% and the NPV was 78.5%. ΔIVC remained a poor predictor (AUC 0.53; 95% CI: 0.38–0.68, for a cutoff point of 47.8%).

In non‐ventilated patients (*n* = 28), ΔVAo decreased from 25.1 % (SD 13.9) to 19.1% (SD 10.2; *p* = 0.023; effect size = −0.48). ΔVCa decreased slightly (21.3%–18.4%; *p* = 0.22), and ΔIVC increased marginally (59.5%–63.2%; *p* = 0.63). Volume responsiveness was observed in 20 cases (71.1%). ΔVAo showed excellent performance with a cutoff of 15.5%, sensitivity of 95%, specificity of 87.5%, AUC of 0.89 (95% CI: 0.73–1), and accuracy of 93%. The LR+ was 7.6, the LR− was −0.08, PPV of 95% and NPV of 88%. ΔVCa had a cutoff of 13.2%, sensitivity of 100%, specificity of 50%, AUC of 0.69 (95% CI: 0.41–0.96), and accuracy of 85%. The LR+ was 2, the LR− −1, the PPV 0.83, and the NPV was 1. ΔIVC had limited utility (AUC 0.6; 95% CI: 0.36–0.85, for a cutoff point of 41%).

**FIGURE 2:**
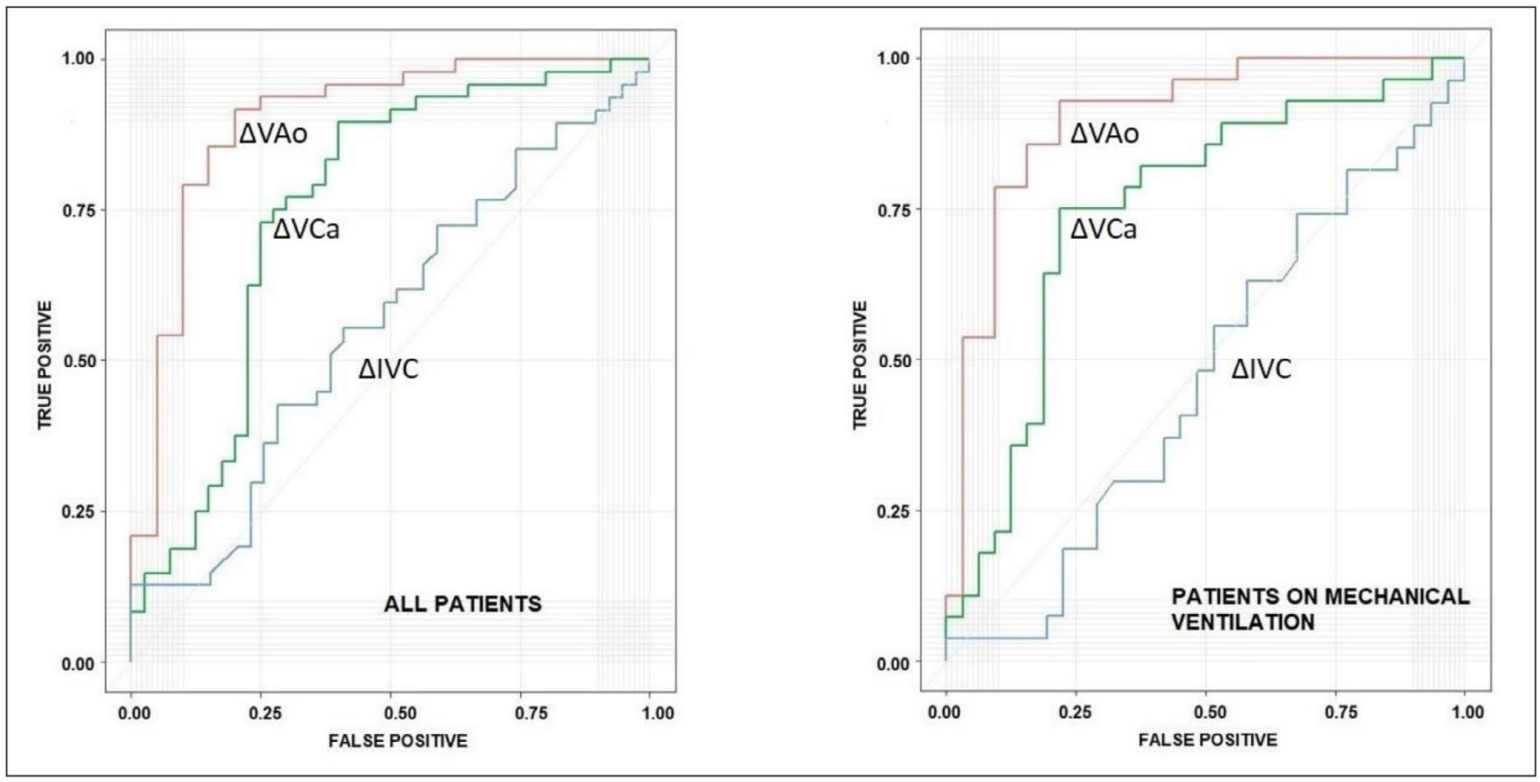
The AUCs for ΔVAo, ΔVCa, and ΔIVC, for all patients and stratified by ventilation status.

Figure 2 illustrates the ROC curves for ΔVAo, ΔVCa, and ΔIVC across the full cohort and stratified by ventilation status.

## 4 DISCUSSION

This study assessed the use of dynamic ultrasound parameters as predictors of fluid responsiveness in critically ill pediatric oncology patients. ΔVAo demonstrated the strongest performance, with sensitivity exceeding 90%, specificity of 80%, and an optimal cutoff of 16.3%. These values suggest substantial clinical utility, and ΔVAo maintained its predictive accuracy across subgroups, including mechanically ventilated and spontaneously breathing patients. In the latter, the cutoff was slightly lower (15.5%) but retained high sensitivity (95%) and specificity (87.5%). Respiratory variation in peak aortic velocity serves as a reliable predictor of fluid responsiveness, as it directly reflects real‐time changes in stroke volume and cardiac output. An increase in preload enhances left ventricular ejection, which is quantifiable as elevated aortic blood flow velocity. Consequently, dynamic fluctuations in velocity indicate whether the heart is functioning on the ascending limb of the Frank–Starling curve, thereby identifying patients likely to exhibit a favorable response to fluid administration (Desgranges et al., 2016).

Fluid responsiveness paradigms differ substantially between adult and pediatric critical care. In adults, a 10%–15% increase in stroke volume after rapidly administered, repeated small boluses is typically used within a preload optimization strategy (Cecconi et al., 2014). Defining fluid responsiveness as a ≥15% increase in cardiac output or cardiac index following a standardized fluid challenge reflects the greater physiological variability, higher heart rates, and increased measurement noise characteristic of children. Cardiac index was chosen as the reference outcome because it is the most commonly used size‐adjusted measure of cardiac performance in pediatric critical care and allows comparison across a wide age and body size range. Although fluid responsiveness is fundamentally related to changes in stroke volume, stroke volume measurement in children is subject to higher relative error and heart rate variability. Using cardiac index mitigates these issues while preserving physiological relevance. This avoids overestimating responsiveness and reduces the risk of unnecessary fluid loading, which is particularly important since children are more vulnerable to fluid overload complications. The present study did not aim to achieve preload optimization through repeated boluses; instead, it sought to assess the diagnostic accuracy of Doppler‐derived indices in predicting a clinically meaningful hemodynamic response to a single fluid challenge, in line with pediatric practice.

Our findings align with previous literature. Gan et al. identified respiratory variation in aortic peak velocity as the only reliable predictor across five studies (Gan et al., 2013). Compared to the meta‐analysis by Sethasathien et al., which reported pooled sensitivity and specificity of 80% and 82%, respectively (Sethasathien et al., 2023), our results show superior sensitivity and comparable specificity. This meta‐analysis included 15 studies with 452 patients in 2023, showing that there are still few patients studied using this method. Notably, the largest prior PICU study involving cancer patients included only 30 individuals, reporting a cutoff of 13.7% with sensitivity and specificity near 80% (Sun et al., 2020).

ΔVCa showed weaker predictive performance overall, particularly in non‐ventilated patients, where specificity dropped to 50% despite 100% sensitivity. However, its consistency in ventilated patients and feasibility in cases where aortic access is limited support its role as a secondary option. Prior studies on ΔVCa, such as those by Souza et al., reported promising results but were limited by small sample sizes (de Souza et al., 2022). A recent study showed that ΔVCa can be comparable to ΔVAo in schoolchildren, but in younger children, it was not reliable enough (Cristiani et al., 2025).

The subgroup of nonresponders warrants particular attention. A failure to increase cardiac output after volume expansion does not necessarily indicate inadequate preload; rather, it may reflect a state in which cardiac output is no longer preload‐dependent. In critically ill children, this phenomenon may be driven by myocardial dysfunction, altered ventriculo‐arterial coupling (a mismatch between ventricular contractility and arterial afterload), increased right ventricular afterload, or microcirculatory impairment, where the microvasculature remains “shunted” or obstructed (Østergaard et al., 2015). Although nonresponders exhibited a statistically significant decrease in heart rate with a moderate effect size—suggesting a physiological response governed by the Frank–Starling principle—there was no corresponding elevation in cardiac output (Pinsky & Payen, 2005).

Several factors further limit the accuracy of doppler‐derived indices in nonresponders. Age is one such factor: ΔVAo appears to have stronger predictive value in children older than 25 months (Wang et al., 2019). Respiratory variability is also greater in pediatric patients, particularly under mechanical ventilation, producing noisy Doppler signals. In non‐sedated children, motion artifacts from crying, agitation, or spontaneous breathing reduce reliability. Probe positioning is more challenging in smaller patients, especially those who are critically ill or mechanically ventilated. Higher heart rates shorten diastolic filling times, complicating waveform interpretation, while smaller stroke volumes make measurements more vulnerable to error. Developmental differences in vascular compliance and autonomic tone further contribute to unpredictable hemodynamic responses. Acoustic windows are often restricted by chest wall anatomy, patient movement, or the presence of tubes and lines (Escribá Alepuz et al., 2024). Although some of these limitations—particularly anatomical and physiological—may be unavoidable, many can be mitigated. With consistent practice and repeated training, clinicians can achieve skill mastery, allowing most technical barriers to be overcome.

Children with cancer face distinctive hemodynamic challenges. Factors such as cancer‐related inflammation, prior exposure to cardiotoxic chemotherapy, anemia, endothelial dysfunction, and recurrent infectious complications can significantly affect cardiovascular reserve and preload responsiveness. These features set them apart from other pediatric ICU populations and contribute to the variability observed in fluid responsiveness (Camargo et al., 2025; Terwoord et al., 2022).

In contrast, ΔIVC demonstrated poor discriminatory power. This finding is particularly relevant since ΔIVC assessment is frequently used in clinical practice in PICUs due to its ease of execution and low training requirements. Consistent with our findings of limited performance, a study by Long et al., which evaluated 33 children with sepsis not on mechanical ventilation, reported that the IVC collapse index demonstrated little utility, with a sensitivity of 0.44, specificity of 0.33, and an AUC of 0.38 (Long et al., 2018). A study by Campos et al., showed in 32 children on mechanical ventilation that the IVC diameter had a sensitivity of 69.2%, specificity of 78.9%, and an AUC of 0.7 (Campos et al., 2024). Our study corroborates the conclusion of the meta‐analysis by Long et al., that respiratory variation in IVC diameter appears to have inadequate test characteristics to support clinical decisions regarding fluid replacement (Long et al., 2017).

Since fluid bolus can have undesirable effects and contribute to fluid overload, we collected other data to evaluate the physiological effects of the volume used in the study (Table 2). Responders showed improved left ventricular ejection fraction and slight reductions in bicarbonate, while nonresponders exhibited increased heart rate and modest lactate reduction. Chloride levels rose significantly in responders. Lung ultrasound revealed increased B‐lines in both groups, more pronounced in nonresponders, though averages remained below thresholds for pulmonary congestion.

The primary goal of volume expansion is to increase cardiac output by increasing venous return pressure. Since the response to volume is inconsistent, the main purpose of a hemodynamic assessment should be to help assess the likelihood of a response, and after the volume is infused, to evaluate its effectiveness and possible deleterious effects (De Backer et al., 2022). Given the potential risks of fluid overload, hemodynamic assessment should guide both the decision to administer fluids and the evaluation of their impact. POCUS offers a comprehensive, noninvasive approach—ΔVAo as a primary tool, ΔVCa as a much less precise alternative, and echocardiography and lung ultrasound for serial monitoring. However, these methods are operator‐dependent and require structured training. To integrate POCUS into standard care for volume administration in critically ill children, the implementation of educational programs and a specific curriculum for residents and attending physicians are essential (Bhargava et al., 2022). Identifying specific cutoff values for ΔVAo and ΔVCa in pediatric oncology patients fills a critical gap in the literature and supports more individualized, evidence‐based fluid management.

Our study has limitations inherent to a single‐center study, which may limit the generalizability of the results. The specific population of children with cancer may differ from other critically ill pediatric populations, and the results may not be directly applicable to all pediatric ICU patients. Because we opted for a single‐examiner assessment protocol, out of respect for patient comfort, we were unable to assess differences between operators. This is a type of study where patient inclusion is difficult, and this is reflected in the small sample sizes present in previous meta‐analyses and published studies. Our sample size did not allow for adequately powered subgroup analyses stratified by age or body weight, particularly in adolescents. However, the use of indexed cardiac output and relative (percentage) Doppler variations was intended to reduce size‐related bias. As a strength, our sample of children with cancer appears to be the largest ever studied for the dynamic markers ΔVAo and ΔVCa, although the subgroup of children not on mechanical ventilation was very small.

Future studies should focus on multicenter validation and on integrating Doppler‐derived indices with echocardiographic assessment of myocardial function and markers of tissue perfusion. Despite methodological challenges, Doppler‐based assessment—particularly ΔVAo—appears to be a promising tool to guide individualized fluid management in critically ill children.

## 5 CONCLUSIONS

Peak aortic velocity variation (ΔVAo) is a reliable predictor of fluid responsiveness in critically ill children with cancer. Carotid flow variation (ΔVCa) may be considered with caution when aortic assessment is not feasible, though with reduced accuracy. Respiratory variation in inferior vena cava diameter (ΔIVC) does not appear to be a useful predictor in this population.

## AUTHOR CONTRIBUTIONS

Bruno S. Camargo: Conceptualization, data curation, formal analysis, investigation, and writing—original draft. Orlei R. de Araujo: Conceptualization, data curation, formal analysis, methodology, supervision, writing—review and editing. Dafne Cardoso B. da Silva: Conceptualization, supervision, writing—review and editing.

## FUNDING INFORMATION

None to declare.

## CONFLICT OF INTEREST STATEMENT

None to declare.

## ETHICS STATEMENT

Ethical approval was granted by the Research Ethics Committee of the Federal University of São Paulo (Certificate No. 68803323.9.0000.5505, approved July 19, 2023), in accordance with Resolution 196/96 of the National Health Council and the principles of the 1975 Declaration of Helsinki.

## Data Availability

The datasets generated and/or analyzed during the current study are not publicly available due to ethical and privacy restrictions involving pediatric patients, but are available from the corresponding author upon reasonable request.
